# Pressure Overload-induced Cardiac Hypertrophy Varies According to Different Ligation Needle Sizes and Body Weights in Mice

**DOI:** 10.5935/abc.20180088

**Published:** 2018-07

**Authors:** Zhen Jia, Zhijun Chen, Hongfei Xu, Malik Akuffu Armah, Peng Teng, Weidong Li, Dongdong Jian, Liang Ma, Yiming Ni

**Affiliations:** 1 Department of Cardio-Thoracic Surgery, The First Affiliated Hospital, School of Medicine, Zhejiang University, Zhejiang - China; 2 Department of Cardio-Thoracic Surgery, Zhoushan Hospital, Zhejiang - China

**Keywords:** Cardiomegaly, Body Weight, Heart Failure, Needles/utilization, Rats

## Abstract

**Background:**

The cardiac hypertrophy (CH) model for mice has been widely used, thereby providing an effective research foundation for CH exploration.

**Objective:**

To research the effects of CH modeling under abdominal aortic constriction (AAC) using different needles and weights in mice.

**Methods:**

Four needles with different external diameters (0.35, 0.40, 0.45, and 0.50 mm) were used for AAC. 150 male C57BL/6 mice were selected according to body weight (BW) and divided into 3 weight levels: 18 g, 22 g, and 26 g (n = 50 in each group). All weight levels were divided into 5 groups: a sham group (n = 10) and 4 AAC groups using 4 ligation intensities (n = 10 per group). After surgery, survival rates were recorded, echocardiography was performed, hearts were dissected and used for histological detection, and data were statistically analyzed, P < 0.05 was considered statistically significant.

**Results:**

All mice died in the following AAC groups: 18g/0.35 mm, 22 g/0.35 mm, 26 g/0.35 mm, 22 g/0.40 mm, and 26 g/0.40 mm. All mice with AAC, those ligated with a 0.50-mm needle, and those that underwent sham operation survived. Different death rates occurred in the following AAC groups: 18 g/0.40 mm, 18 g/0.45 mm, 18 g/0.50 mm, 22 g/45 mm, 22 g/0.50 mm, 26 g/0.45 mm, and 26 g/0.50 mm. The heart weight/body weight ratios (5.39 ± 0.85, 6.41 ± 0.68, 4.67 ± 0.37, 5.22 ± 0.42, 4.23 ± 0.28, 5.41 ± 0.14, and 4.02 ± 0.13) were significantly increased compared with those of the sham groups for mice with the same weight levels.

**Conclusion:**

A 0.45-mm needle led to more obvious CH than did 0.40-mm and 0.50-mm needles and caused extraordinary CH in 18-g mice.

## Introduction

Cardiac hypertrophy (CH) is a compensatory pathological change that is usually induced by pressure overload (PO), neurohumoral abnormality, and the effects of cytokines. It is characterized by cardiomyocyte hypertrophy and interstitial hyperplasia, and it results in an enlarged heart and thickening of the heart walls. Clinically, CH is involved in the development of many diseases, such as valvular disease, hypertension, arterial stenosis, and primary myocardial hypertrophy. If these diseases develop at their own pace, then cardiac function (CF) will gradually decompensate, leading to heart failure (HF), which severely lowers the quality of life and increases the mortality rate. Therefore, CH is a widespread concern and has been explored at the molecular level by researchers. Due to the high genomic homology between mice and humans, an established CH model for mice has been widely used in animal experiments, thereby providing an effective research foundation for CH exploration.

Currently, PO-induced CH is a common way to establish the model. Abdominal aortic constriction (AAC) is highly recommended by researchers because of the high success rate and the ability to perform surgery without the need for thoracotomy or a ventilator. However, the modeling effects with different ligating intensities for certain body weights (BWs) have not yet been reported. Therefore, we used 3 frequently used mice BWs (18 g, 22 g, and 26 g) and 4 different needle sizes (0.35, 0.40, 0.45, and 0.50 mm) to establish the CH model for each weight level for AAC, summarized the survival rates, and evaluated the CH effects.

## Methods

### Animal groups and handling

One-hundred fifty male C57BL/6 wild-type mice were obtained from the Shanghai SLAC Laboratory Animal Co. Ltd (Shanghai, China). All animals were treated and cared for in accordance with the *Guide for the Care and Use of Laboratory Animals* (National Institutes of Health, Washington, DC, 1996). Experimental protocols were approved by our Institutional Animal Care and Use Committee of Zhejiang University (Hangzhou, China). Mice were selected according to weights of approximately 18 g (range, 17.3-18.7 g), 22 g (range, 20.8-23.0 g), and 26 g (range, 25.1-27.0 g), and they were divided into the following 3 weight levels: 18 g (18.0 ± 0.3 g; n = 50), 22 g (22.0 ± 0.6 g; n = 50), and 26 g (26.1 ± 0.5 g; n = 50). All weight levels were divided using sortition randomization method to create a sham group (n = 10) and 4 AAC groups according to ligating intensities (0.35, 0.40, 0.45, and 0.50mm; n = 10 per group). Regarding BW, no significant differences were found among the 5 groups for each weight level (Table S1), and the preoperative BWs of mice that died and those that survived were not significant (Table S2).

Mice were anesthetized with 4% chloralhydrate (0.1ml/1g BW, intraperitoneal injection). When the mice did not respond when their toe was pinched, the limbs were fixed on the operating board in the supine position and the skin was prepared by shaving and disinfection with alcohol. Sterile gauze was placed on the right side of the abdomen and a ventrimesal incision approximately 1.5 cm was created starting from the xiphoid. The skin was fixed with a spreader and the viscera was pulled out gently with a swab and placed on the gauze. Then, the abdominal aorta was isolated using a blunt dissection technique with curved microforceps under a microscope. A 6-0 silk suture was snared and pulled back around the aorta 1mm above the superior mesenteric artery. A 2-mm blunt acupuncture needle (external diameters: 0.35 mm, 0.40 mm, 0.45 mm, and 0.50 mm; Huatuo; Suzhou Medical Appliance Factory, Suzhou, China; criterion number GB2024-1994) was then placed next to the aorta. The suture was tied snugly around the needle and the aorta. The needle was removed immediately after ligation, the viscera were replaced, the peritoneum and skin were sutured, and the mice were allowed to recover. Aortic ligation was omitted only for the sham group. After surgery, the ears were cut to differentiate the mice. Then, mice were placed in an incubator at 30ºC until they woke, and they were returned to their cages. Survival status was recorded daily. To observe the physical development of mice under different conditions, BW differences before surgery and at week 8 post-surgery were calculated as the change in BW.

### Echocardiography imaging

After post-surgery weeks 4 and 8, mice were weighed and anesthetized with 4% chloralhydrate and placed on a warming pad after skin preparation. Transthoracic 2-dimensional (2D) echocardiography was performed using the GE Vivid E9 Ultrasound echocardiographic system (General Electric Company, Fairfield, CT, USA) with the GE 9L probe (8-MHz linear array transducer; General Electric Company). M-mode parasternal long-axis scans of the left ventricle at the mitral chordae level were used to quantify the interventricular septum thickness at end-diastole (IVSd), interventricular septum thickness at end-systole (IVSs), left ventricular internal dimension at end-diastole (LVIDd), left ventricular internal dimension at end-systole (LVIDs), left ventricular posterior wall thickness at end-diastole (LVPWd), left ventricular posterior wall thickness at end-systole (LVPWs), ejection fraction (EF), and fractional shortening (FS). All mice were tested using the same parameters.

### Heart weight, heart weight/body weight, and heart weight/tibial length

After echocardiographic analysis at 8 weeks post-surgery, mice were sacrificed by cervical dislocation and the hearts were dissected. Then, atrial and vascular tissues were snipped carefully, leaving the ventricles. The hearts were rinsed with phosphate-buffered saline (PBS), drained by gently squeezing on absorbent paper, weighed, photographed under natural light, and fixed in 4% paraformaldehyde. The tibial lengths (TLs; mean value of the bilateral tibia) were recorded. Heart weight (HW), BW, and TL were measured, and the HW/BW ratio and HW/TL ratio were calculated to evaluate the hypertrophic response to PO.

### Histological examination of the heart

Extracted hearts were fixed in 4% paraformaldehyde for 24h and dehydrated. After routine histologic procedures, the hearts were embedded in paraffin and cut into 4-µm sections. Sections were stained with hematoxylin and eosin (HE) and picrosirius red (PSR). Cardiac cross-sections were captured at 20 × microscopic views from HE sections, and 5 thicknesses of the left ventricle in each view were selected in systematic sampling, and measured using Image-Pro Plus 6.0 (Media Cybernetics, Inc., Rockville, MD, USA). Then, the mean values were calculated. Cardiomyocyte morphological changes were captured at 400×microscopic views from HE sections. Interstitial and/or perivascular collagen depositions were captured at 200×microscopic views under standard lights. Collagen was stained red using PSR, thereby indicating fibrosis. At least 6 views were selected in a blinded manner, and each photograph was analyzed to reveal the ratio of red collagen to the entire tissue area using Image-Pro Plus 6.0. Then, the mean values were calculated.

### Statistical Analysis

SPSS 17.0 statistical software (SPSS Inc., Chicago, IL, USA) was used for all statistical analyses. The Kolmogorov-Smirnov (K-S) test was used to verify the normality of the quantitative variables as appropriate. Data are presented as mean ± standard deviation (SD). One-way ANOVA and post-hoc Tukey tests were used to evaluate differences between groups. p < 0.05 was considered statistically significant.

## Results

### Excessive AAC may lead to death

We monitored mice deaths after surgery according to acute heart failure (AHF) criteria. Data (Table 1) showed that all deaths occurred within 5 days, and a high incidence of death occurred during the initial 24h post-surgery.

**Table 1:** Mice deaths after surgery

	Needles (mm) for 18 g				Needles (mm) for 22 g				Needles (mm) for 26 g		
	0.35	0.40	0.45		0.35	0.40	0.45		0.35	0.40	0.45
0-24 h	10	4	1		10	7	1		10	8	3
24 h-3 d	0	0	0		0	2	0		0	1	2
3 d-5 d	0	0	1		0	1	2		0	1	1

There were no mice deaths in the AAC0.50-mm group or the sham group. Deaths were recorded during 3 time periods (0-24h, 24h-3d, and 3-5 d); 54 deaths occurred within 0-24h post-surgery. The total number of deaths was 65.

### AAC increases cardiac dimensions and reduces cardiac function

Echocardiography was performed at the end of post-operative weeks 4 and 8. At week 4 post-surgery, data (Table 2) showed a trend of heart enlargement for mice with AAC, including thickening of the ventricular wall and an increase in chamber dilation; however, differences in EF and FS were not significant, indicating that changes in the heart structure did not have a pronounced effect on cardiac function at that time point. At week 8 post-surgery, the trend of heart enlargement continued; however, the EF and FS values for the AAC groups decreased significantly. This change in cardiac function from week 4 to week 8 was consistent with systolic function beginning to be markedly affected at week 4 after PO surgery.

**Table 2:** Echocardiographic outcomes of 18-g, 22-g, and 26-g mice

		18 g/0.40 mm (n = 6)		18 g/0.45 mm (n = 8)		18 g/0.50 mm (n = 10)		18 g/Sham (n = 10)		22 g/0.45 mm (n = 7)		22 g/0.50 mm (n = 10)		22 g/Sham (n = 10)		26 g/0.45 mm (n = 4)		26 g/0.50 mm (n = 10)		26 g/Sham (n = 10)
**Week 4**																				
IVSd		0.92 ± 0.05*		0.96 ± 0.05*		0.86 ± 0.05		0.81 ± 0.04		0.82 ± 0.06		0.83 ± 0.04		0.78 ± 0.04		0.84 ± 0.03		0.80 ± 0.04		0.80 ± 0.04
IVSs		1.12 ± 0.05		1.24 ± 0.15*		1.08 ± 0.10		1.05 ± 0.04		1.06 ± 0.07*		1.00 ± 0.04		0.98 ± 0.03		1.15 ± 0.08		1.11 ± 0.07		1.08 ± 0.08
LVIDd		3.21 ± 0.31		3.33 ± 0.26		3.00 ± 0.16		3.08 ± 0.28		3.04 ± 0.20		3.01 ± 0.17		3.00 ± 0.19		3.40 ± 0.11*		3.11 ± 0.15		2.96 ± 0.22
LVIDs		2.13 ± 0.26		2.37 ± 0.26*		2.03 ± 0.11		2.09 ± 0.21		1.92 ± 0.13		1.98 ± 0.14		2.07 ± 0.18		2.24 ± 0.18*		2.02 ± 0.13		1.93 ± 0.14
LVPWd		0.94 ± 0.04*		1.01 ± 0.08*		0.87 ± 0.04		0.81 ± 0.09		0.85 ± 0.04		0.87 ± 0.05*		0.81 ± 0.07		0.90 ± 0.06		0.89 ± 0.05		0.87 ± 0.06
LVPWs		1.13 ± 0.06		1.24 ± 0.06*		1.08 ± 0.09		1.04 ± 0.03		1.04 ± 0.04		1.00 ± 0.07		1.04 ± 0.10		1.27 ± 0.04*		1.12 ± 0.07		1.10 ± 0.05
EF %		70.7 ± 3.8		66.0 ± 3.9		67.9 ± 3.5		68.5 ± 2.6		72.1 ± 4.5		68.3 ± 3.7		70.6 ± 3.6		70.5 ± 5.4		71.2 ± 3.1		73.1 ± 4,.7
FS %		34.5 ± 2.4		31.2 ± 2.1*		34.5 ± 2.3		34.3 ± 2.4		36.9 ± 2.3		35.7 ± 3.0		36.7 ± 2.8		36.3 ± 2.6		37.5 ± 2.3		36.6 ± 3.2
**Week 8**																				
IVSd		0.93 ± 0.08*		0.97 ± 0.05*		0.88 ± 0.04		0.83 ± 0.07		0.99 ± 0.06*		0.91 ± 0.02*		0.84 ± 0.07		0.93 ± 0.08		0.91 ± 0.06		0.85 ± 0.06
IVSs		1.18 ± 0.20		1.33 ± 0.14*		1.15 ± 0.07		1.10 ± 0.10		1.26 ± 0.07*		1.16 ± 0.10*		1.04 ± 0.08		1.19 ± 0.10		1.17 ± 0.11		1.14 ± 0.07
LVIDd		3.34 ± 0.24		4.12 ± 0.34*		3.30 ± 0.41		3.14 ± 0.15		3.26 ± 0.13		3.15 ± 0.13		3.23 ± 0.15		3.50 ± 0.12*		3.29 ± 0.16		3.20 ± 0.15
LVIDs		2.13 ± 0.11		3.02 ± 0.27*		2.21 ± 0.40		1.94 ± 0.18		2.25 ± 0.11		2.11 ± 0.14		2.19 ± 0.12		2.50 ± 0.15*		2.33 ± 0.26		2.14 ± 0.15
LVPWd		0.96 ± 0.08*		1.03 ± 0.08*		0.93 ± 0.04*		0.84 ± 0.08		0.99 ± 0.05*		0.96 ± 0.04*		0.90 ± 0.06		1.02 ± 0.07*		0.96 ± 0.04*		0.90 ± 0.04
LVPWs		1.23 ± 0.08*		1.35 ± 0.13*		1.20 ± 0.07*		1.07 ± 0.07		1.22 ± 0.06*		1.14 ± 0.07		1.07 ± 0.08		1.30 ± 0.08*		1.13 ± 0.05*		1.04 ± 0.06
EF %		64.7 ± 4.6*		60.9 ± 2.4*		67.6 ± 4.7*		75.5 ± 5.5		63.3 ± 3.0*		67.7 ± 3.3*		74.2 ± 3.2		62.8 ± 2.6*		67.5 ± 5.3*		73.1 ± 2.9
FS %		33.2 ± 3.0*		29.4 ± 1.9*		35.8 ± 4.3*		41.0 ± 5.5		32.9 ± 1.6*		35.5 ± 2.4*		40.8 ± 3.1		31.0 ± 3.2*		32.7 ± 3.8*		36.9 ± 2.5

IVSd: interventricular septum thickness at end-diastole; IVSs: interventricular septum thickness at end-systole; LVIDd: left ventricular internal dimension at end-diastole; LVIDs: left ventricular internal dimension at end-systole; LVPWd: left ventricular posterior wall thickness at end-diastole; LVPWs: left ventricular posterior wall thickness at end-systole; EF: ejection fraction; FS: fractional shortening. The cardiac dimensions (inclding IVSd, IVSs, LVIDd, LVIDs, LVPWd, and LVPWs) (mm) and functional indices (inclding EF and FS) changes measured by echocardiography. Data were statistically analyzed and presented as the mean ± SD. At week 4, cardiac dimensions for the AAC groups significantly increased compared with the sham groups (*p < 0.05); at week 8 , more cardiac dimensions significantly increased, and that EF and FS values for the AAC groups all decreased significantly compared to those of the sham groups for 3 weight levels (*p < 0.05).

### AAC increases HW, HW/BW, and HW/TL ratio

Generally, the increased HW, HW/BW, and HW/TL ratio are the three main indicators of CH. In our study, as shown in Table 3, we found that AAC significantly increased HW and caused a significantly higher HW/BW ratio and HW/TL ratio compared to the sham groups for all weight levels. The HW, HW/BW, and HW/TL values for the AAC0.45 mm groups were significantly higher than those for the AAC0.50 mm groups. These HW-related indices for the 18 g/0.45 mm groups were even significantly higher than those for the 18 g/0.40 mm groups.

**Table 3:** Heart weight–related indices of 18-g, 22-g, and 26-g mice

		18 g/0.40 mm (n = 6)		18 g/0.45 mm (n = 8)		18 g/0.50mm (n = 10)		18 g/Sham (n = 10)		22 g/0.45 mm (n = 7)		22 g/0.50 mm (n = 10)		22 g/Sham (n = 10)		26 g/0.45 mm (n = 4)		26 g/0.50 mm (n = 10)		26 g/Sham (n = 10)
HW		136.5 ± 22.3*		170.0 ± 21.4**		124.0 ± 9.9*		103.5 ± 7.0		137.1 ± 7.4**		115.5 ± 7.6*		104.3 ± 7.4		153.5 ± 4.8**		114.3 ± 5.1*		103.2 ± 5.6
HW/BW		5.39 ± 0.85*		6.41 ± 0.68**		4.67 ± 0.37*		3.86 ± 0.18		5.22 ± 0.42**		4.23 ± 0.28*		3.62 ± 0.26		5.41 ± 0.14**		4.02 ± 0.13*		3.59 ± 0.16
HW/TL		63.8 ± 10.3*		74.4 ± 9.3**		57.6 ± 4.6*		47.8 ± 3.6		59.6 ± 3.3**		54.2 ± 3.8*		48.3 ± 3.9		65.6 ± 1.3**		49.2 ± 2.6*		44.1 ± 2.8

HW: heart weight; BW: body weight; TL: tibial length. HW(mg), HW/BW(mg/g), and HW/TL(mg/cm) were measured and calculated from AAC and sham groups of 3 BW levels. Data are presented as the mean ± SD.

* Compared to the sham group in the same BW level, the heart weight–related indices of AAC groups increased significantly (p < 0.05).

** Compared to the rest groups in the same BW level, the heart weight–related indices of the AAC 0.45-mm group increased significantly (p < 0.05).

### AAC leads to cardiomyocyte hypertrophy and increases collagen depositions

**Figure 1:**
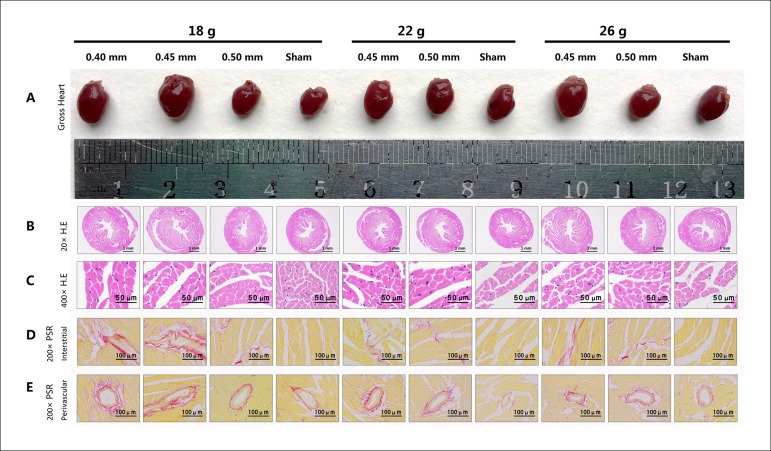
Cardiomyocyte hypertrophy and collagen deposition histological examination. (A) Gross hearts under natural light. (B) The 20 × microscopic views of HE sections. (C) The 400 × microscopic views of HE sections. (D) Representative 200×microscopic views under standard lights of PSR sections in the interstitial space. (E) Representative 200 × microscopic views under standard lights of PSR sections in the perivascular space. Fibrosis is presented as red in the PSR sections.

For mice undergoing AAC surgery, the hearts demonstrated different degrees of enlargement (Figure 1A), enlargement of the papillary muscles, and thickening of the ventricular walls (Figure 1B). Wall thickening increased significantly compared with that of the sham group (Table 4). The sham groups showed normal architecture of the cardiomyocytes compared with the AAC groups. Pathological changes including enlarged, disarrayed, and eosinophilic cardiomyocytes and cardiomyocytes rich in cytoplasm and trachychromatic and pantomorphic nuclei were observed in each of the AAC groups (Figure 1C). Scattered collagen depositions in the interstitial and perivascular spaces were observed in the sham groups. In comparison, in some AAC groups, a larger quantity and wider range of red deposits were observed in the interstitial space (Figure 1D), and thickened collagen was observed in the perivascular space, especially in the external vascular wall (Figure 1E). Statistical analysis indicated that the AAC group had a significantly greater collagen area than the sham group (Table 5). These results imply that AAC is capable of inducing PO-induced CH and fibrosis.

**Table 4:** Thickness of the left ventricle (mm) based on weight and needle size

Weight	0.40 mm	0.45 mm	0.50 mm	Sham
18 g	1.81 ± 0.30*	1.86 ± 0.17*	1.59 ± 0.09*	1.27 ± 0.07
22 g		1.69 ± 0.24*	1.55 ± 0.19*	1.22 ± 0.14
26 g		1.82 ± 0.30*	1.59 ± 0.22*	1.34 ± 0.07

Data are presented as the mean ± SD (n = 5).

* p < 0.05 represents a significant difference between the abdominal aortic constriction (AAC) and sham groups.

**Table 5:** Percentage of collagen deposition in the left ventricle based on weight and needle size

Weight	0.40 mm	0.45 mm	0.50 mm	Sham
18 g	5.8 ± 2.2*	8.9 ± 1.3*	5.1 ± 1.3*	2.6 ± 1.0
22 g		5.2 ± 1.6*	4.9 ± 1.5*	2.5 ± 0.9
26 g		6.1 ± 1.0*	5.3 ± 1.8*	3.1 ± 0.8

Data are presented as the mean ± SD (n = 6).

* p < 0.05 represents a significant difference between the abdominal aortic constriction (AAC) and sham groups.

### AAC may restrict physical development

Analysis showed that with AAC 0.45 mm, BW significantly increased in 18-g mice compared to 22-g and 26-g mice (Table 6), indicating that the 18-g groups had higher development potential. In the 18-g mice groups, data showed that the value of 18 g/0.40 mm was significantly lower than that of the 18 g/0.45 mm and 18 g/sham groups, and that there were no significant differences between the 18 g/0.45 mm and 18 g/sham groups (Table 7), indicating that the 18 g/0.45 mm group had nearly normal physical development. Development of the 18 g/0.40 mm group was limited.

**Table 6:** Body weight changes with AAC under 0.45 mm needle

	18 g/0.45 mm (n = 8)	22 g/0.45 mm (n = 7)	26 g/0.45 mm(n = 4)
Change in BW(g)	8.4 ± 0.8*	4.4 ± 0.8	2.4 ± 0.3

BW: body weight; AAC: abdominal aortic constriction. Data are presented as the mean ± SD.

* p < 0.05 represents a significant difference between the 18 g/0.45 mm group and the 22 g/0.45 mm and 26 g/0.45 mm groups.

**Table 7:** Body weight and BW changes in 18-g mice

	18 g/0.40 mm (n = 6)	18 g/0.45 mm (n = 8)	18 g/0.50 mm (n = 10)	18 g/Sham (n = 10)
BW before surgery (g)	18.1 ± 0.4	18.1 ± 0.3	18.0 ± 0.4	17.9 ± 0.4
BW at week 8 (g)	25.3 ± 0.4*	26.5 ± 0.9	26.6 ± 0.8	26.8 ± 0.9
BW change (g)	7.2 ± 0.6*	8.4 ± 0.8	8.6 ± 0.6	8.8 ± 0.9

BW: body weight. BW changes of 18-g mice before and after surgery for 8 weeks. Data are presented as the mean ± SD.

* p < 0.05 represents a significant difference between the 18 g/0.40 mm group and the rest groups after surgery.

## Discussion

In this study, we performed AAC according to 4 different ligating intensities for mice of 3 different weight levels to evaluate the survival rates of mice and CH induced by PO under different conditions. This is the first study showing that CH diversities exist among groups under different ligations and BW.

AAC is widely used in the modeling of CH induced by PO in mice. Needle ligation is usually used, and the efficiency of modeling is highly dependent on ligation intensity. Nevertheless, excessive constriction will lead to death,^1^ and our research findings (Table 1) demonstrated this point. In this study, a 0.35-mm needle caused the death of all mice in the 3 weight levels, and the 0.40-mm needle caused the death of all mice in 22-g and 26-g groups. Contrarily, all mice with AAC that underwent surgery with a 0.50-mm needle or sham operation survived. Mice in the other groups had different mortality rates. Regarding the selection of needles for the BW ranges of this study, a needle smaller than 0.35mm in diameter caused stronger constriction and death. However, a needle larger than 0.50 mm in diameter did not alternatively affect the survival rate, but it did reduce the efficiency of CH because of the reduced PO from weaker constriction. This is why we chose needles between 0.35 mm and 0.50 mm.

Death can occur after AAC. Undoubtedly, AAC increases cardiac afterloading. To cope with the additional biodynamics, the heart exerts a series of adaptive changes, including activation and hypertrophy of cardiomyocytes and hyperplasia of the extracellular matrix.^2^ This compensational mechanism maintains cardiac output (CO) effectively for a period of time while maintaining the survival of the organism; it is also the basis for the establishment of the CH model. However, when the sudden afterloading is out of the range of cardiomyocyte adjustment, the bloodstream will be limited and cause constriction, resulting in AHF. AHF is typically characterized by rapid changes in heart failure (HF) symptoms.^3^ Sato et al.^4^ considered the incidence of death within 5 days as an assessment criterion of AHF. AHF could moderately or markedly improve by the second day if effectively controlled. AHF leads to high ventricular pressure, and high ventricular pressure leads to high pulmonary blood pressure, thus leading to pulmonary congestion, which is one of the causes of death after AAC.^5^ Liao et al.^6^ suggested that cardiogenic pneumo-edema is the main cause of postoperative death for PO mice. Additionally, arrhythmia may occur as part of the electrophysiological changes,^7^ and cardiomyocyte sarcomeres may be disordered during the pathological changes.^8^ These are all severe threats to the survival rate after AAC. Our record of mice death times (Table 1) showed the phenomenon of all deaths occurring within 5 days. A high incidence of death occurred during the initial 24h, which is in accordance with the aforementioned AHF criteria. In addition, there is a positive correlation between CO and BW;^9,10^ therefore, compared with the low-weight mice, high-weight mice require more CO and will have cardiac afterloading that is more increased than that of low-weight mice with the same aortic constriction. Results of the current study (Table 1) indicate that higher-weight mice had poorer tolerance for AAC, which is reflected in their mortality rates. Regarding mice with AAC that underwent ligation with a 0.40-mm needle, all mice in the 22-g and 26-g groups died. However, 6 out of 10 mice survived in the 18-g group.

The diagnosis of CH usually depends on changes in cardiac function and morphology.^11^ Echocardiography can be performed in vitro noninvasively during the first assessment of CH, and it is especially used to monitor changes in cardiac function.^12^ We performed echocardiographic examinations of mice at the end of week 4 and week 8 post-surgery. Data (week 4 data in Table 2) showed that at the end of week 4, the phenomena of thickened ventricular walls, enlarged ventricular chambers, and decreased cardiac functions were emerging in each AAC group compared with the sham groups, and this diversity was consistent with the characteristic cardiac changes that occur with chronic pressure overload.^13,14^ These trends became more pronounced at the end of week 8 (week 8 data in Table 2), when EF and FS, which represent cardiac function, were significantly lower compared with the sham groups. CH also increased HW. In our study, the HW, HW/BW ratio, and HW/TL ratio for the AAC groups were significantly increased (Table 3). Cardiac remodeling is the most typical pathological change of CH, including cardiomyocyte hypertrophy and the extracellular matrix increases.^15^ Our histological results showed increased external diameters and ventricular thickness in gross hearts and cross-sections under AAC (Figures 1A and B). HE staining of the AAC groups displayed the hypertrophic pathology of cardiomyocytes and nuclei (Figure 1C). PSR staining of the AAC groups displayed extensive collagen depositions (Figure 1D), particularly in the perivascular space (Figure 1E). Statistical analysis showed that the thickness of the left ventricle (Table 4) and the percentage of collagen deposition (Table 5) were significantly increased in the AAC groups compared to the sham group. Regarding the formation of collagen, Kuwahara et al.^16^ indicated that cardiac fibroblasts are activated on day 3 after PO, and that the neoformative fibrous tissues mainly affect the diastolic function rather than the systolic function during the initial 4 weeks. Then, excessive myocardial fibrosis is implicated in systolic dysfunction because of its more intensive traction, and cardiac function begins to deteriorate significantly. Regarding EF and FS values for the AAC groups (Table 2), the downward trends from week 4 to week 8 conform to this theory.

Choosing the proper needle is critical for establishing the CH model. Based on these results, we found that all mice with AAC died when a 0.35-mm needle was used for ligation for all 3 weight levels and when a 0.40-mm needle was used for ligation for the 22-g and 26-g groups; therefore, these 5 groups of weight-needle pairings were clearly unsuitable for use. The 18g/0.40mm group had obvious CH compared with the sham group, and its survival rate was acceptable (6 out of 10). However, it should still be excluded because the 18 g/0.45 mm group showed more obvious CH and higher survival rates (8 out of 10) (Table 1, Table 3). The 0.45-mm and 0.50-mm needles are available for all 3 weight levels, but both can result in definite myocardial hypertrophy. However, the values of the HW, HW/BW ratio, and HW/TL ratio for the AAC mice when using the 0.45-mm needle were significantly higher than those when using a 0.50-mm needle for each weight level (Table 3). Therefore, for all 3 weight levels of our study, a CH model can be established using a 0.50-mm needle and the survival rate of the mice will not be threatened. However, a 0.45-mm needle leads to more effective CH model, and higher mortality than the 0.50-mm needle.

Normally, with the PO-induced CH model, thinner needles creates more severe aortic stenosis and lead to more pronounced CH, and vice versa. However, we observed an interesting phenomenon: the CH level of the 18 g/0.45 mm group was abnormally significantly higher than that of the 18 g/0.40 mm group (18-g mice in Table 3). Regarding the analysis of BW data with AAC (Table 6), the changes in BW in 18-g mice during weeks 0 to 8 were significantly higher than those for the 22-g and 26-g mice, indicating that 18-g mice have greater potential for physical development after surgery and that physical development is often accompanied by organ development.^17^ Therefore, the heart of 18-g mice also has greater development potential. For the same weight level, the BW change of the 18 g/0.45 mm group during weeks 0 to 8 was significantly higher than that of the 18 g/0.40 mm group (BW change in Table 7). As mentioned, BW is positively related to CO; therefore, perhaps the greater ligation limited CO in the 18 g/0.40 mm group, which also limited physical development and organ development, including development of the heart. At the end of week 8, there was no significant difference in BW for the 18g/0.45mm group and 18g/sham groups; both had significantly higher BW than the 18 g/0.40 mm group (BW at week 8 in Table 7). The 0.45-mm needle had no obvious limits in18-g mice, but the BW advantage for the 18 g/0.45 mm group compared to the 18 g/0.40 mm group depends on greater CO and requires more hypertrophic myocardium for support. So, to establish CH models for AAC in mice that have developmental potential, such as 18-g mice, there may be a special ligating intensity region that can cause more obvious CH than the two adjacent regions. However, this phenomenon must comprise multiple factors and is worth further study.

## Conclusion

We established CH models using 4 ligation needle sizes and 3 weights for mice. Data showed that both of 0.45-mm and 0.50-mm needles lead to CH. However, 0.45mm needle brings more effective model and causes obvious CH in 18-g mice.

## Supplementary Materials

**Table S1:** Mice body weights before surgery

Weight	0.35 mm	0.40 mm	0.45 mm	0.50 mm	Sham
18 g	18.0 ± 0.4	18.0 ± 0.3	18.1 ± 0.3	18.0 ± 0.4	17.9 ± 0.4
22 g	22.1 ± 0.4	22.0 ± 0.6	21.9 ± 0.6	22.2 ± 0.7	22.1 ± 0.6
26 g	26.0 ± 0.7	26.1 ± 0.5	26.0 ± 0.5	25.9 ± 0.3	26.3 ± 0.5

No significant differences were found among the 5 groups according to pair-wise comparisons of each weight level (p > 0.05); therefore, body weight could be considered one index for the same weight level. Data are presented as the mean ± SD (g) (n = 10). Body weights did not differ significantly from each other (p > 0.05).

**Table S2:** Mice body weights before abdominal aortic constriction

Weight	Survival	Death
18 g	18.1 ± 0.3 (n = 24)	18.0 ± 0.3 (n = 16)
22 g	22.1 ± 0.7 (n = 17)	22.0 ± 0.5 (n = 23)
26 g	25.9 ± 0.4 (n = 14)	26.1 ± 0.6 (n = 26)

Retrospective data showed that the difference between weight at death and survival were not significant for each weight level (p > 0.05), indicating that individual weight differences for the same weight level had no influence on postoperative death. Data are presented as mean ± SD (g).

## References

[1] Zhao CZ, Zhao XM, Yang J, Mou Y, Chen B, Wu HD (2016). Inhibition of farnesyl pyrophosphate synthase improves pressure
overload induced chronic cardiac remodeling. Sci Rep.

[2] Frey N, Olson EN (2003). Cardiac hypertrophy: the good, the bad, and the
ugly. Annu Rev Physiol.

[3] Alla F, Zannad F, Filippatos G (2007). Epidemiology of acute heart failure syndromes. Heart Fail Rev.

[4] Sato N, Lam CS, Teerlink JR, Greenberg BH, Tsutsui H, Oh BH (2017). Evaluating the efficacy, safety, and tolerability of serelaxin
when added to standard therapy in Asian patients with acute heart failure:
design and rationale of RELAX-AHF-ASIA trial. J Card Fail.

[5] Yamamoto K, Ohishi M, Katsuya T, Ito N, Ikushima M, Kaibe M (2006). Deletion of angiotensin-converting enzyme 2 accelerates pressure
overload-induced cardiac dysfunction by increasing local angiotensin
II. Hypertension.

[6] Liao Y, Bin J, Asakura M, Xuan W, Chen B, Huang Q (2012). Deficiency of type 1 cannabinoid receptors worsens acute heart
failure induced by pressure overload in mice. Eur Heart J.

[7] Martinez-Rubio A, Schwammenthal Y, Schwammenthal E, Block M, Reinhardt L, Garcia-Alberola A (1997). Patients with valvular heart disease presenting with sustained
ventricular tachyarrhythmias or syncope: results of programmed ventricular
stimulation and long-term follow-up. Circulation.

[8] Massengill MT, Ashraf HM, Chowdhury RR, Chrzanowski SM, Kar J, Warren SA (2016). Acute heart failure with cardiomyocyte atrophy induced in adult
mice by ablation of cardiac myosin light chain kinase. Cardiovasc Res.

[9] Foppa M, Arora G, Gona P, Ashrafi A, Salton CJ, Yeon SB (2016). Right ventricular volumes and systolic function by cardiac
magnetic resonance and the impact of sex, age, and obesity in a
longitudinally followed cohort free of pulmonary and cardiovascular disease:
the Framingham Heart Study. Circ Cardiovasc Imaging.

[10] Karimian S, Stein J, Bauer B, Teupe C (2016). Impact of severe obesity and weight loss on systolic left
ventricular function and morphology: assessment by 2-dimensional
speckle-tracking echocardiography. J Obes.

[11] Elliott P, McKenna WJ (2004). Hypertrophic cardiomyopathy. Lancet.

[12] Zhao B, Wang S, Chen J, Ji Y, Wang J, Tian X (2017). Echocardiographic characterization of hypertrophic cardiomyopathy
in Chinese patients with myosin-binding protein C3 mutations. Exp Ther Med.

[13] Hunter JJ, Chien KR (1999). Signaling pathways for cardiac hypertrophy and
failure. N Engl J Med.

[14] Huang CK, Chen BY, Guo A, Chen R, Zhu YQ, Kutschke W (2016). Sildenafil ameliorates left ventricular T-tubule remodeling in a
pressure overload-induced murine heart failure model. Acta Pharmacol Sin.

[15] Tsotetsi OJ, Woodiwiss AJ, Netjhardt M, Qubu D, Brooksbank R, Norton GR (2001). Attenuation of cardiac failure, dilatation, damage, and
detrimental interstitial remodeling without regression of hypertrophy in
hypertensive rats. Hypertension.

[16] Kuwahara F, Kai H, Tokuda K, Kai M, Takeshita A, Egashira K (2002). Transforming growth factor-beta function blocking prevents
myocardial fibrosis and diastolic dysfunction in pressure-overloaded
rats. Circulation.

[17] Minior VK, Levine B, Ferber A, Guller S, Divon MY (2017). Nucleated red blood cells as a marker of acute and chronic fetal
hypoxia in a rat model. Rambam Maimonides Med J.

